# Maxillary unicystic ameloblastoma: a case report

**DOI:** 10.1186/s13104-016-2260-7

**Published:** 2016-10-18

**Authors:** Zana Agani, Vjosa Hamiti-Krasniqi, Jehona Recica, Mergime Prekazi Loxha, Fisnik Kurshumliu, Aida Rexhepi

**Affiliations:** 1 Department of Oral Surgery, University Dentistry Clinical Center of Kosova, Prishtina, Republic of Kosovo; 2 Advanced Dentistry Clinic “Identity”, Prishtina, Republic of Kosovo; 3 Department of Maxillofacial Surgery, University Clinical Center of Kosova, Prishtina, Republic of Kosovo; 4 Department of Pathology, University Clinical Center of Kosova, Prishtina, Republic of Kosovo; 5 Department of Pedodontics, University Dentistry Clinical Center of Kosova, Prishtina, Republic of Kosovo

**Keywords:** Ameloblastoma, Unicystic ameloblastoma, Tooth impaction, Enucleation

## Abstract

**Background:**

Ameloblastoma is a benign epithelial odontogenic tumor. It is often aggressive and destructive, with the capacity to attain great size, erode bone and invade adjacent structures. Unicystic ameloblastoma is a rare odontogenic lesion, with clinical, radiographic and gross features of jaw cysts. The lesion histologically shows typical ameloblastomatous epithelium lining part of the cyst cavity with or without and/or mural tumor growth. Unicystic ameloblastoma usually presents in posterior mandibular ramus region, while it is rare and atypical in posterior maxillary region. .

**Case presentation:**

We report a case of 16 year old Kosovar male, Albanian ethnicity, who presented with a swelling located in right maxillary region. Clinical examination revealed a painless swelling extending from the maxillary right central incisor to the maxillary right first molar tooth. Panoramic radiograph disclosed a well corticated unilocular radiolucent lesion approximately 5 × 5 cm in diameter which was in contact with the roots of the teeth present inferiorly and with the maxillary sinus superiorly. Maxillary right canine impaction was noted and unerupted lateral incisor tooth was present inside the radiolucency. Preoperative diagnosis of the lesion was made as dentigerous cyst based on the age of the patient, location of the swelling, clinical and radiographic findings, but the unicystic ameloblastoma was also taken into consideration. The patient was treated by surgical enucleation of the lesion and extraction of lateral incisor tooth which was present inside the lesion. The histopathological examination of the lesion revealed confirmed finding for unicystic ameloblastoma mural form. No recurrence was observed in 1 year follow-up.

**Conclusions:**

Maxillary region is considered a rare and atypical location for unicystic ameloblastoma. We emphasize the importance of differential diagnosis of an odontogenic lesion with common clinical and radiological features that will impact the treatment planning and follow up. As oral health providers we should be aware that the unilocular radiolucencies may be unicystic ameloblastoma.

## Background

Ameloblastoma is a local invasive tumor which originates from remnants of the dental lamina and odontogenic epithelium and it accounts for only 1 % of all oral tumors [1, 2]. Based on the World Health Organization (WHO) classification of head and neck tumours, there are four forms of ameloblastomas: multicystic, peripheral, desmoplastic and unicystic ameloblastomas [3].

Unicystic ameloblastoma (UA) as a distinct entity was first described by Robinson and Martinez in 1977 [4]. UA refers to those cystic lesions with clinical, radiographic, or gross features of a jaw cyst, with which they are usually differentially diagnosed, but on histological examination the UA shows a typical ameloblastomatous epithelium lining part of the cyst cavity, with or without luminal and/or mural tumor growth [5]. UA accounts for about 6 % of ameloblastomas, and 50 % of cases occur in the second decade of life, more often in mandible than in maxilla [6]. The response of UA to enucleation or curettage is more favorable than the solid or multicystic ameloblastomas [5].

The purpose of this article is to present a rare case report of UA in atypical location into the right anterior and premolar maxillary region together with two impacted teeth.

## Case presentation

A 16-year-old Kosovar male, of Albanian ethnicity referred to our department with the chief complaint of painless swelling on the right cheek. The patient described painless swelling 3 months before visit as the initial observation, but has shown the enlargement in the last few weeks. Medical history data of the patient revealed no systemic disease or other health problems.

Facial asymmetry was present on the right side on clinical extraoral examination.

The skin overlying the swelling was normal. The extraoral swelling was well circumscribed, painless and approximately 5 × 5 cm in size. The consistency was hard and without fluctuation.

Intraoral examination revealed a painless swelling in the right maxillary vestibule extending from the maxillary right central incisor to the maxillary right first molar. The intraoral swelling was firm, non tender, covered with normal mucous membrane.

**Fig. 1:**
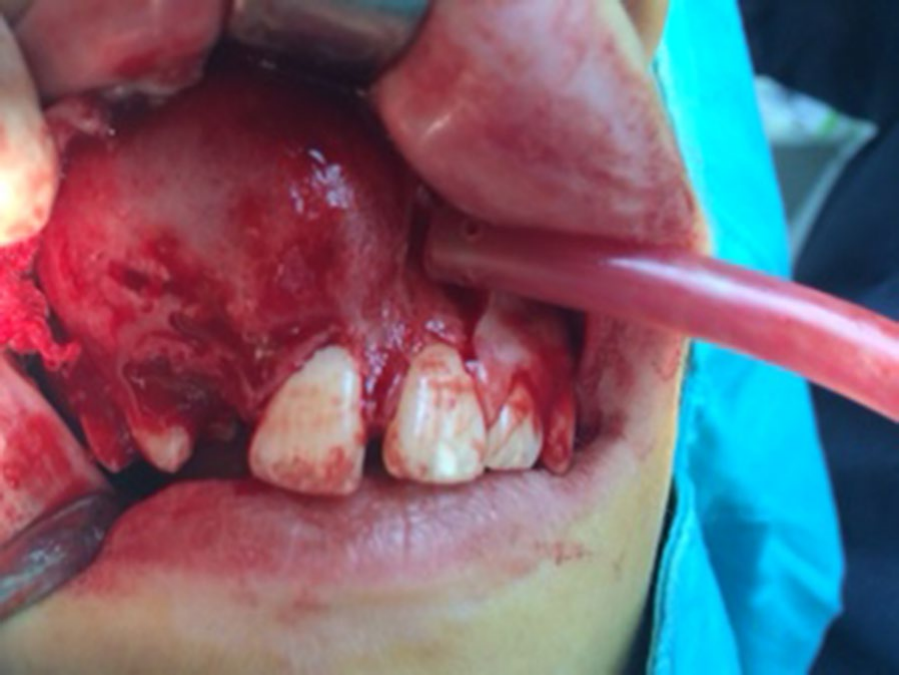
The egg shell cracking present in the right maxillary vestibulum arising from central incisor 11 and distally to the first molar tooth of the same site

**Fig. 2:**
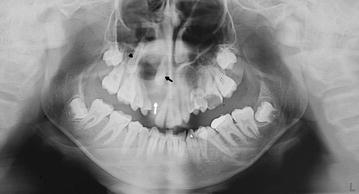
Panoramic radiograph showing large lesion (*white arrow*) in right maxilla associated with impaction of lateral incisor (*black arrow*) and canine tooth (*arrowhead*) of same site

Egg shell cracking was present buccally but not palatally (Fig. 1). Aspiration revealed thick juicy yellow liquid and cholesterol crystals were visible. Panoramic radiograph revealed a unilocular radiolucent lesion extending from the maxillary right central incisor to the maxillary right first molar, in contact with the roots of the teeth present inferiorly, and to the maxillary sinus superiorly. Maxillary right canine tooth was displaced posteriorly most probably by the cystic pressure and the unerupted lateral incisor was present inside the radiolucency (Fig. 2). The vitality of teeth 11, 14, 15, with roots in close relation to the lesion was positive.

**Fig. 3:**
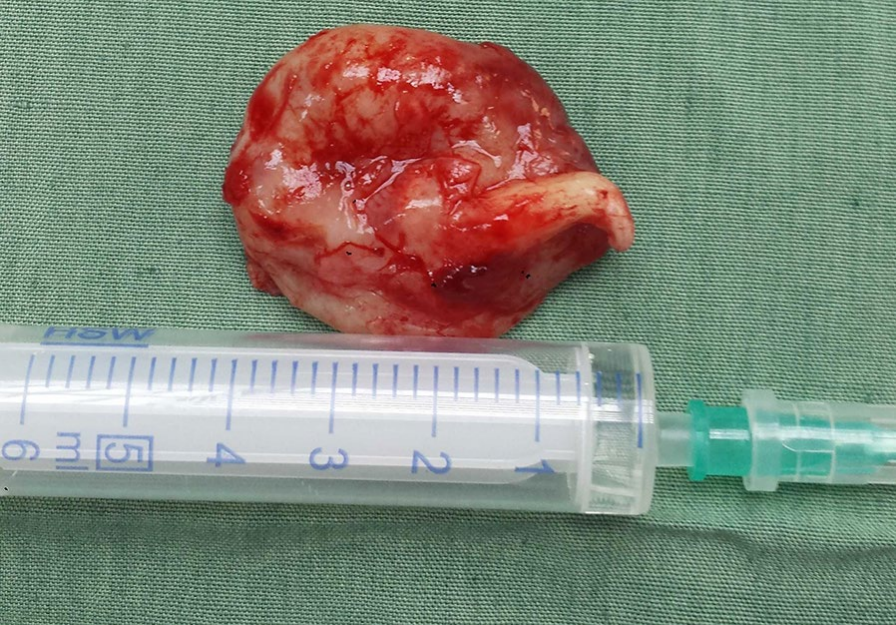
The enucleated lesion measured dimension with lateral incisor inside the lesion

**Fig. 4:**
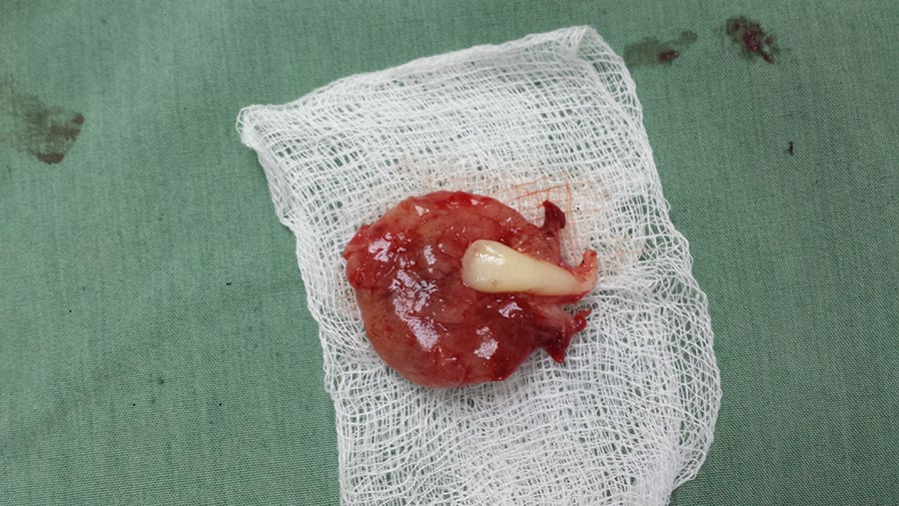
The enucleated lesion measured dimension with lateral incisor inside the lesion

Preoperative diagnosis of the lesion was made as dentigerous cyst based on the age of the patient, location of the swelling, impacted right canine and unerupted lateral incisor located inside the lesion, aspirated thick juicy yellow liquid and visible cholesterol crystals, but the UA was also taken into consideration. The surgical operation including total enucleation of the cystic lesion together with impacted tooth was made (Figs. 3, 4). After removing the lesion along with the impacted lateral incisor tooth and after measuring it, the lesion was approximately 4 cm in length (Fig. 4). The wound was tamponated with gauge which was removed periodically for 3 days from the postoperative second day. The specimen was sent for pathological examination.

**Fig. 5:**
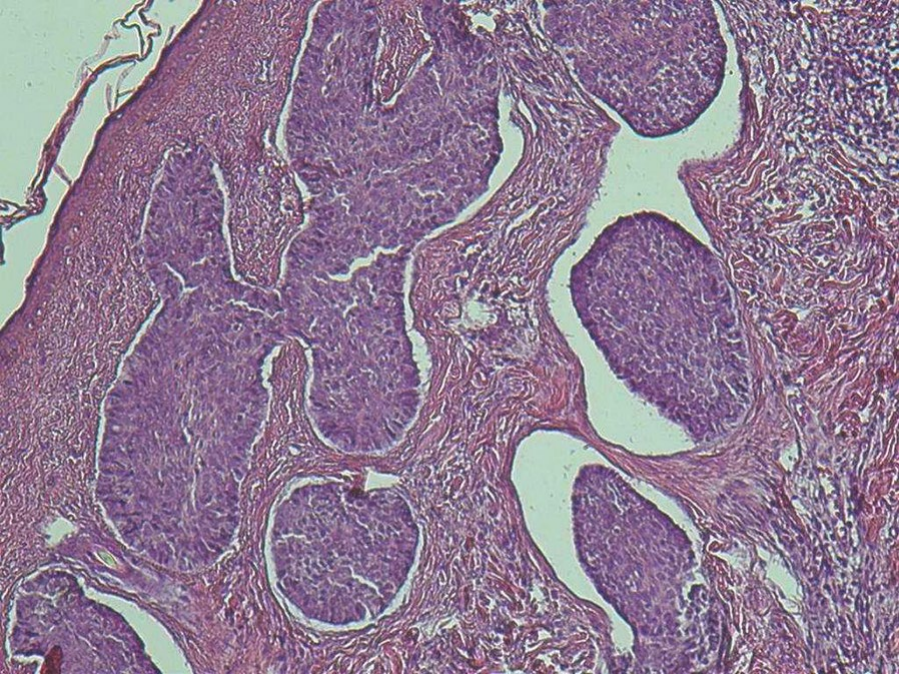
Infiltrating islands of atypical basaloid cells with peripheral palisading. Separation artifact of peritumoral stroma is evident. Microscopic picture showing ameloblastic epithelium in the* right-hand side* of the picture, in contrast to gingival squamous epithelium to the left (Hematoxylin and Eosin ×10 magnification)

The pathological examination revealed UA, mural form. Infiltrating islands of atypical basaloid cells with peripheral palisading were present. Separation artifact of peritumoral stroma was evident (Fig. 5).

The nature of the tumor was explained to the patient and we advised the patient to regard regular follow-up visits. There were no signs of recurrence since 2 years after the operation.

## Discussion

UA is a rare type of ameloblastoma, accounts for about 6 % of all ameloblastomas. It affects mandible more often than maxilla and in about 50 % of the cases occur in the second decade of life [7]. It is presented more commonly in the mandible than in the maxilla in the ratio of 13:1. The tumor is observed in mandibular-ramus region, while posterior region of maxilla is considered to be rare and atypical [6].

The lesion is usually found in association with the crowns of mandibular third molar teeth, but can be seen also in interradicular, periapical and edentulous regions as well [8]. In our case it is associated with the maxillary lateral incisor tooth. It is presented as a painless swelling, facial asymmetry, tooth impaction, tooth displacement, mobility, or tooth resorption. On radiographic imaging the unilocular lesion with well defined sclerotic borders is seen [9]. The differential diagnosis of UA should include keratocystic odontogenic tumor, residual cyst, central fibroma, central giant cell granuloma and dysplastic fibrosis [1].

Ackermann et al. (1988) [9] and Robinson and Martinez (1977) [4] argued that as the epithelium of odontogenic cysts and ameloblastomas have a common ancestry, a transition from a nonneoplastic cyst to a neoplastic one could be possible, even though it occurs infrequently.

Radiographically there are 2 main patterns: Unilocular and multilocular [10, 11].

Based on histological examination, to diagnose a lesion as unicystic ameloblastoma, the minimum criteria is the demonstration of presence of a single cystic sac lined by odontogenic ameloblastomatous epithelium which is seen only in focal areas [12].

There are different classifications of unicystic ameloblastoma. Based on the clinicopathologic study of 57 cases of unicystic ameloblastoma, Ackerman’s classification into three histologic groups is as follows:

I. Luminal UA (tumor confined to the luminal surface of the cyst);
II. Intraluminal/plexiform UA (nodular proliferation into lumen without infiltration of tumor cells into connective tissue wall); and
III. Mural UA (invasive islands of ameloblastomatous epithelium in the connective tissue wall not involving the entire epithelium) [9].

According to this classification, our case study belongs to Group III.

There is another grouping by Philipsen and Reichart [13] which describes the forms of UA as follows:

Subgroup 1. Luminal UA;

Subgroup 1.2. Luminal and intraluminal;

Subgroup 1.2.3. Luminal, intraluminal and intramural; and

Subgroup 1.3. Luminal and intramural.

UA is considered to be a less aggressive form of ameloblastomas that can be successfully removed by simple enucleation or other less aggressive surgery [14].

The use of Carnoy’s solution to decrease the risk of recurrence after conservative surgical treatment of UA’s was initially suggested by Stoelinga and Bronkhorst in 1988 [15]. Also it is advocated that vigorous curettage of the bone should be avoided because it may implant foci of ameloblastoma more deeply in bone [16]. The recurrence rate for UA’s after conservative surgical treatment (curettage or enucleation) is generally reported 10–20 % [17] and on average, <25 % [18]. This is considerably less than 50–90 % recurrence rates which are noted after the conventional curettage of solid or multicystic ameloblastomas [17, 19]. Lau and Samman [20] reported recurrence rates of 3.6 % for resection, 30.5 % for enucleation alone, 16 % for enucleation followed by Carnoy’s solution application, and 18 % by marsupialisation followed by enucleation, where the lesion is reduced in size.

## Conclusions

Every unilocular radiolucency of the jaw should be closely monitored and examined since UA shares significant clinical and radiographic similarities with odontogenic cysts and tumors. Neither the incisional biopsy may be able to reflect the true nature of the lesion nor the aspirational cytology. Long-term follow-up is mandatory because of the recurrence risk of unicystic ameloblastoma, which may occur after a long time.

## Abbreviations

UA: unicystic ameloblastoma
WHO: World Health Organisation

## References

[1] Piscevic A, Gavric M, Sjerobabin I. Maksilofacialna Hirurgija, Izdavacka Agencija “Draganic”, Beograd, 1995. pp. 344–6.

[2] Li TJ, Kitano M (1997). Reviewing the unicystic ameloblastoma: a clinicopathologically distinct entity. Oral Med Pathol..

[3] Kramer IRH, Pindborg JJ, Shear M (1992). The World Health Organization histological typing of odontogenic tumours. A commentary on the 2nd Edition. Cancer.

[4] Robinson L, Martinez MG (1977). Unicystic ameloblastoma: a prognostically distinct entity. Cancer.

[5] Gardner DG (1984). A pathologist’s approach to the treatment of ameloblastoma. J Oral Maxillofac Surg.

[6] Philipsen HP, Reichard PA (1998). Unicystic ameloblastoma. A review of 193 cases from literature. Oral Oncol.

[7] Ramesh RS, Manjunath S, Ustad TH, Pais S, Shivakumar K (2010). Unicystic ameloblastoma of the mandible—an unusual case report and review of literature. Head Neck Oncol.

[8] Isacsson G, Andersson L, Forsslund H, Bodin I, Thomsson M (1986). Diagnosis and treatment planning of unicystic ameloblastoma. Int J Oral Maxillfac Surg.

[9] Ackermann GL, Altini M, Shear M (1988). The unicystic ameloblastoma: a clinicopathological study of 57 cases. J Oral Pathol.

[10] Eversole LR, Leider AS, Strub D (1984). Radiographic characteristics of cystogenic ameloblastoma. Oral Surg Oral Med Oral Pathol.

[11] Paikkatt VJ, Sreedharan S, Kannan VP (2007). Unicystic ameloblastoma of the maxilla: a case report. J Indian Soc Pedod Prev Dent.

[12] Zainab C, Vandana S, Pal US, Pankaj S (2011). Unicystic ameloblastoma: a diagnostic dilemma. Natl J Maxillofac Surg..

[13] Chana JS, Chang YM, Wei FC, Shen YF, Chan CP, Lin HN (2004). Segmental mandibulectomy and immediate free fibula osteoseptocutaneous flap reconstruction with endosteal implants: an ideal treatment method for mandibular ameloblastoma. Plast Reconstr Surg.

[14] Handa H, Bailoor DN, Naidu G, Shrivastava K, Raghuvanshi V (2013). Unicystic Ameloblastoma of mandible. Aggressive treatment A myth or a need. Case report and extensive review of literature. IOSR J Dent Med Sci.

[15] Stoelinga PJW, Bronkhorst FB (1988). The incidence, multiple presentation and recurrence of aggressive cysts of the jaws. J Craniomaxillofac Surg.

[16] Li TJ, Kitano M, Arimura K, Sugihara K (1998). Recurrence of unicystic ameloblastoma: a case report and review of the literature. Arch Pathol Lab Med.

[17] Neville BW, Damm DD, Allen CM, et al. Oral and Maxillofacial Pathology, 3rd edn. St. Louis, Mo: Saunders; 2009.

[18] Gardner DG, Corio RL (1984). Plexiform unicystic ameloblastoma. A variant of ameloblastoma with a low-recurrence rate after enucleation. Cancer.

[19] Ameerally P, McGurk M, Shaheen O (1996). Atypical ameloblastoma: report of 3 cases and a review of the literature. Br J Oral Maxillofac Surg.

[20] Lau SL, Samman N (2006). Recurrence related to treatment modalities of unicystic ameloblastoma: a systematic review. Int J Oral Maxillofac Surg.

